# Neutrophil extracellular traps: a novel contributor to vascular calcification in chronic kidney disease

**DOI:** 10.3389/fimmu.2025.1639166

**Published:** 2025-09-29

**Authors:** Yan Wang, Wenjun Li, Yufeng Liang, Jianxin Wan

**Affiliations:** ^1^ Department of Nephrology, Blood Purification Research Center, The First Affiliated Hospital, Fujian Medical University, Fuzhou, China; ^2^ Department of Nephrology, Mengchao Hepatobiliary Hospital of Fujian Medical University, Fuzhou, China; ^3^ Department of Nephrology, The Second Hospital of Longyan, Fujian, China; ^4^ Fujian Clinical Research Center for Metabolic Chronic Kidney Disease, The First Affiliated Hospital, Fujian Medical University, Fuzhou, China; ^5^ Department of Nephrology, National Regional Medical Center, Binhai Campus of the First Affiliated Hospital, Fujian Medical University, Fuzhou, China

**Keywords:** CKD, vascular calcification, neutrophil extracellular traps, MMP12, COMP, machine learning.

## Abstract

**Background:**

Among patients with chronic kidney disease (CKD), vascular calcification significantly contributes to cardiovascular health issues, though the underlying molecular mechanisms remain unclear. Recent research highlights neutrophil extracellular traps (NETs) as critical mediators of vascular damage and pro-calcific processes.

**Methods:**

We obtained transcriptomic data from the NCBI GEO database for CKD rodent models and identified differentially expressed genes, selected genes using machine learning, functional enrichment, profiling of immune infiltration, transcription factor (TF) activity prediction and drug–gene interaction analysis.

**Results:**

Our analysis revealed 36 NET-related genes with differential expression, and 19 were confirmed by the RobustRankAggreg method. Among them, *Mmp12* and *Comp* emerged as the most consistently selected diagnostic markers across five machine learning algorithms, exhibiting excellent predictive performance (AUC > 0.95). These genes were enriched in neutrophil chemotaxis, ECM remodeling, and PI3K-Akt-mTOR signaling pathways. Immunohistochemistry confirmed NET deposition in calcified arteries of rat, and quantitative PCR and Western blot validated key NRGs expression in CKD rat aortae.

**Conclusion:**

Our results demonstrate that NET-related genes may contribute to CKD-associated vascular calcification in rodent models. Specifically, this work provides evidence for a potential mechanistic link between NET biology and vascular calcification in CKD, thereby offering insights into immune-vascular interactions and raising the possibility of exploring NET-targeted approaches to mitigate vascular damage.

## 1 Introduction

Chronic kidney disease (CKD) is a major contributor to global health challenges, causing an estimated 5 to 10 million deaths every year. It involves progressive kidney failure and markedly elevated cardiovascular risk, which continue to be the primary cause of death in individuals with CKD (1, 2). The increased cardiovascular risk in CKD is multifactorial and partly attributable to CKD-specific pathophysiological processes, including vascular calcification. Unlike age-related intimal calcification, CKD predominantly promotes medial arterial calcification—a tightly regulated and dynamic process driven by a combination of systemic and local disturbances (3–6). Among the central mechanisms is disordered mineral metabolism, particularly hyperphosphatemia, hypocalcemia, and secondary hyperparathyroidism (7–9).

CKD is characterized by persistent low-level inflammation, fostering a vascular environment conducive to calcification. Inflammatory agents such as IL-6 and TNF-α promote the recruitment of immune cells, cause oxidative damage, and disrupt endothelial function, speeding up vascular remodeling and mineral buildup. This chronic inflammatory milieu accelerates extracellular matrix (ECM) degradation, facilitates vascular smooth muscle cell (VSMC) phenotypic switching, and initiates calcific lesion formation (10, 11). Furthermore, ECM remodeling driven by matrix metalloproteinases (MMPs), including MMP-2 and MMP-9, disrupts vascular elasticity and exposes nucleation sites for mineral deposition (12, 13). Although progress has been made in understanding the mineral dysregulation in CKD, the exact molecular mediators that link inflammation, matrix remodeling, and calcification are still incompletely elucidated.

In various chronic illnesses, neutrophil extracellular traps (NETs) are being increasingly identified as significant factors in sterile inflammation and vascular injury. NETs are crucial in thromboinflammatory processes and are now regarded as major contributors to immunothrombosis (14–17). NETs have also been associated with the progression of non-infectious diseases, such as autoimmune diseases, cancer, thrombosis, and chronic inflammatory conditions (18). Recent studies have emphasized the potential link between neutrophil activation, NET formation, and increased cardiovascular risk in CKD patients. These patients exhibit elevated serum levels of cytokines that induce NETs, persistent low-grade inflammation, and heightened expression of neutrophil activation markers in the bloodstream (19–22). However, the molecular pathways of NET production in CKD and their role in CKD-related cardiovascular risk remain poorly understood. In particular, NET-related genes are differentially expressed in calcified aortic tissue from uremic mice, however, how these genes connect to major transcriptional and signaling pathways, and whether they could be utilized as diagnostic indicators or therapeutic targets. Hence, a systematic exploration of NET-related gene expression profiles, immune and regulatory networks in CKD-related vascular calcification is warranted. Such insights could illuminate novel mechanisms underlying the CKD–cardiovascular axis and offer mechanistic rationale for targeted interventions.

This research focused on mapping the molecular profile of NET-related genes in aortic calcification caused by CKD through a combined bioinformatics and machine learning strategy. By analyzing transcriptomic data from murine CKD models and incorporating differential expression analysis, immune infiltration profiling, transcription factor (TF) activity prediction, and drug–gene interaction mapping, we identified candidate diagnostic biomarkers, mechanistic regulators, and potential therapeutic targets. Our research provides new insights into how NETs contribute to vascular calcification and lays the groundwork for further mechanistic studies and practical interventions in CKD.

## 2 Materials and methods

### 2.1 Dataset collection and NETs-related gene acquisition

Gene expression data were retrieved from the National Center for Biotechnology Information’s Gene Expression Omnibus (GEO) public repository. To ensure relevance to our research focus on NETs-mediated vascular calcification in CKD, we used “vascular calcification”, “CKD” and “chronic kidney disease” as key words. The selection of GSE146638 (rat, n=5 CKD, n=5 control) and GSE159832 (mouse, n=2 CKD, n=2 control) was driven by their direct alignment with our experimental model. NETs-related genes were collected from previous studies (23, 24) and the GeneCards database (Relevance score ≥10). Homologous gene conversion was conducted using the homologene function in Homologene (version 1.4.68) to map human NETs-related genes to mouse orthologs, as well as rat genes to mouse orthologs.

### 2.2 Differential gene expression analysis

Differential expression analysis was performed using the limma package (version 3.60.6) (25) on log2 transformed FPKM values. Genes with *p-value* < 0.05 and |log2FC| > 1.5 were considered significantly differentially expressed. Volcano plots were visualized using ggplot2 (version 3.5.1).

### 2.3 RRA-based DEG integration

RobustRankAggreg (RRA) analysis was conducted using the RobustRankAggreg package (version 1.2.1) (26) to integrate DEGs from both GEO datasets. Genes with an RRA score ≤ 0.3 were retained as robust NETs-related candidates. Heatmaps were generated using the ComplexHeatmap package (version 2.20.0).

### 2.4 Functional enrichment analysis

Gene ontology (GO: BP, CC, MF), KEGG, Reactome, and WikiPathway enrichment analyses were performed using the Metascape platform (27) with a threshold of *p value* < 0.01 and minimum enrichment score > 1.5. Plots were visualized using ggplot2 (version 3.5.1) and ggh4x (version 0.3.0).

### 2.5 Protein–protein interaction network analysis

PPI networks were constructed via STRING (version 12.0), with a minimum interaction score of 0.4. K-means clustering was applied, and networks were visualized in Cytoscape (version 3.9.1). Hub genes were identified using the cytoHubba plugin (version 0.1) based on Degree centrality.

### 2.6 Gene set enrichment analysis and gene set variation analysis

GSEA was performed using clusterProfiler (version 4.12.6) (28) based on gene expression ranks from both DEGs and gene-correlation analyses. Enrichment was evaluated against KEGG and Hallmark gene sets. Enrichment scores for NETs-related gene sets were calculated using the GSVA package (version 1.44.2) (29). Differences between CKD and control groups were assessed using Wilcoxon tests.

### 2.7 Machine learning-based feature gene selection

Before applying the machine learning algorithms for feature gene selection, we first performed data standardization using the z-score method (i.e., applying the scale function in R, which centers each feature to mean = 0 and scales to standard deviation = 1) on the expression matrix (FPKM). This step ensures comparability across robust NETs-related genes. Subsequently, five machine learning algorithms—LASSO regression, random forest (RF), recursive feature elimination, gaussian mixture modeling (GMM), and support vector machines (SVM)—were used for feature selection.

1. LASSO: glmnet (version 4.1-8) with 10-fold cross-validation to determine the optimal λ and select non-zero weight genes.
2. RF: RandomForest (version 4.7-1.2), selecting top 10 genes by importance scores.
3. RFE: caret (version 7.0-1), iteratively removing low-impact genes to determine the optimal subset.
4. GMM: mclust (version 6.1.1) to cluster genes based on expression variability.
5. SVM-RFE: e1071 (version 1.7-16) + caret for recursive ranking and feature extraction.

Intersection analysis was performed using VennDiagram (version 1.7.3) and UpSetR (version 1.4.0). Candidate diagnostic genes were visualized by expression boxplots (ggpubr, version 0.6.0) and ROC curves using pROC (version 1.18.2).

### 2.8 Immune infiltration analysis

Immune cell proportion in GSE146638 was evaluated using the CIBERSORT package (version 0.1.0) (30). Group comparisons (using Wilcoxon test) and gene–immune correlations (Spearman method) were performed.

### 2.9 Gene co-expression network analysis (GeneMANIA)

The GeneMANIA database was used to construct PPI and functional association networks of diagnostic genes and their related partners, including co-expression, shared pathways, and protein domain similarity.

### 2.10 Transcription factor activity prediction

NETact (31) was employed to infer TF activity from normalized RNA-seq expression matrices (log2(FPKM + 1)). A transcriptional regulatory network was constructed based on TF–target relationships. TF activity scores were computed using expression correlation and topological features. Heatmaps were plotted to display TF activity differences between CKD and control groups.

### 2.11 Drug–gene interaction network

Candidate therapeutic drugs targeting the diagnostic genes were retrieved using the DGIdb. A drug–gene interaction network was constructed and visualized in Cytoscape (version 3.9.1).

### 2.12 Upstream–downstream inference via Bayesian network (CBNplot)

Enrichment analyses were performed using clusterProfiler (version 4.12.6). Bayesian regulatory networks were inferred using CBNplot to identify hierarchical relationships among biological processes and predict upstream and downstream genes involved in CKD progression.

### 2.13 Validation of feature genes in CKD-MBD rat model

6–8 weeks old Sprague-Dawley (SD) rats(n=3) received adenine at 250 mg/kg plus a 1.8% high-phosphorus diet to induce a CKD-MBD model. Eight weeks later, thoracic aortas were collected for gene and protein analyses.

Immunohistochemical (IHC) Staining. Paraffin sections (3 µm) of thoracic aorta from three different CKD rats were deparaffinized, antigen-retrieved, and incubated with anti-MPO (1:500, Servicebio, 4°C, overnight).

### 2.14 Statistical analysis

All statistical analyses were conducted in R (version 4.4.1). A *p* value of < 0.05 was considered statistically significant.

## 3 Results

### 3.1 Identification of NETs-associated genes in CKD with aortic calcification

**Figure 1:**
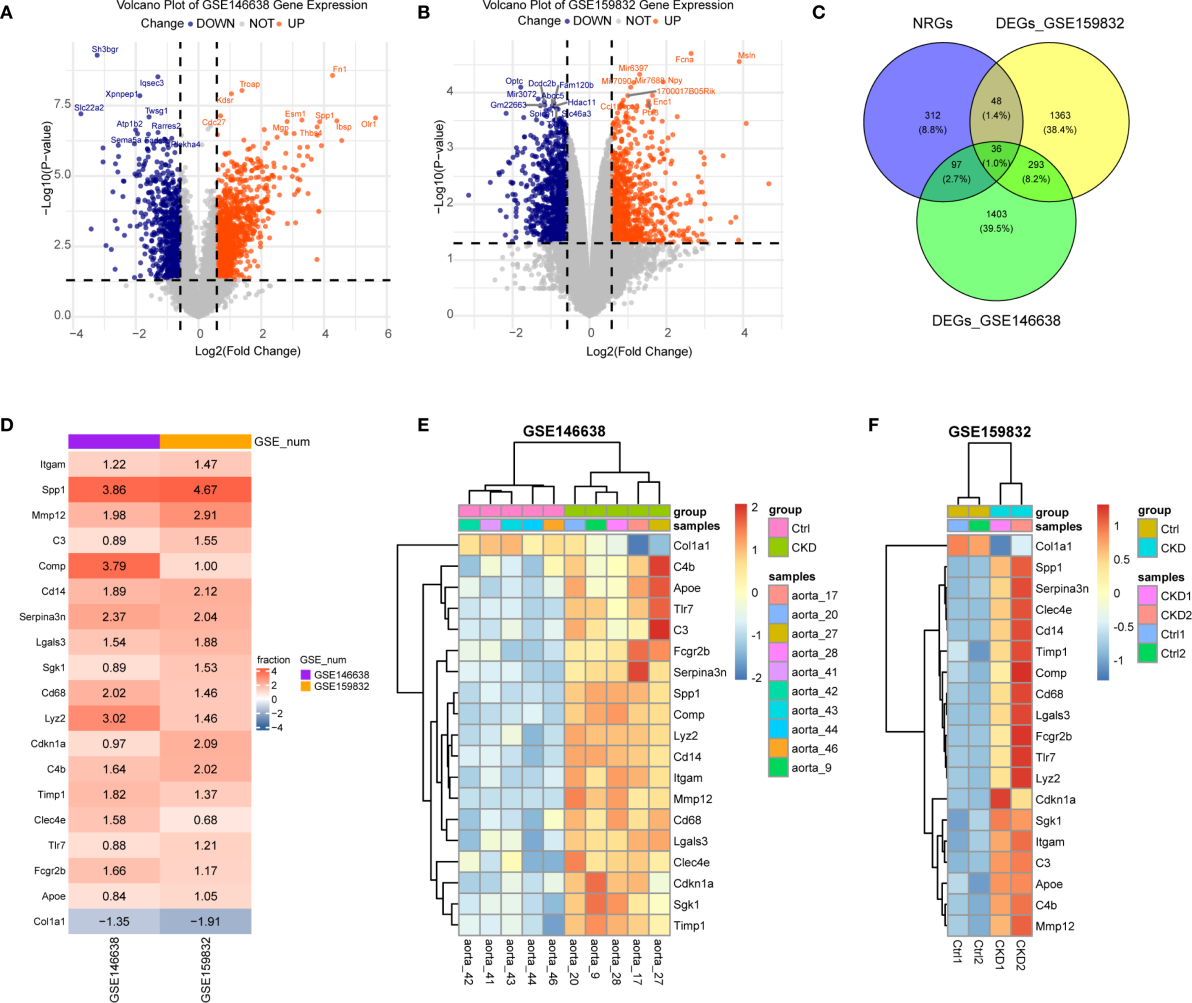
Identification of differentially expressed NET-related genes (NRGs) in CKD-associated vascular calcification. **(A, B)** Volcano plots of differentially expressed genes (DEGs) in GSE146638 **(A)** and GSE159832 **(B)**. Red dots, up-regulated genes; blue dots, down-regulated genes. **(C)** Venn diagram showing the overlap between DEGs and NRGs. **(D)** Robust rank aggregation (RRA) reveals consistently dysregulated NRGs in GSE146638 and GSE159832. Red, up-regulated; blue, down-regulated. **(E, F)** Expression levels of RRA-selected NRGs in CKD versus control groups for GSE146638 **(E)** and GSE159832 **(F)**.

To assess how NETs contribute to vascular calcification in the context of CKD, we examined RNA-seq data from rodent models mimicking arterial calcification observed in this condition of subtotal nephrectomy, which mimic arterial calcification under both atherosclerotic and medial calcification conditions. For this analysis, two datasets were retrieved from the GEO database. Differential gene expression analysis between CKD and control groups revealed 1,058 upregulated and 771 downregulated genes in GSE146638 (**Figure 1A**), and 780 upregulated and 960 downregulated genes in GSE159833 (**Figure 1B**), with a significance threshold of |log2 fold change| > 0.58 and adjusted p-value < 0.05.

We compiled a broad set of NET-associated genes (NRGs) by aggregating entries from peer-reviewed studies and public databases. Specifically, 69 NRGs were obtained from Zhang et al., 137 from Wu et al., and 459 additional candidates were retrieved from the GeneCards database using a relevance score ≥10. After removing duplicates and performing ortholog mapping, a total of 494 unique NRGs were compiled. By intersecting this list with the DEGs extracted from the CKD datasets, 36 overlapping genes were identified as being associated with both NETs and CKD-related aortic calcification (**Figure 1C**).

In order to further refine this gene set, we implemented the RobustRankAggreg (RRA) algorithm (version 1.2.1), which ranks genes based on consistent differential expression across datasets. This analysis yielded 414 consistently dysregulated genes (RRA score < 0.3). Cross-referencing these with the previously identified NETs-related DEGs resulted in 19 robust candidate genes: *Itgam, Tlr7, Sgk1, C3, Clec4e, Fcgr2b, Lyz2, Spp1, Timp1, C4b, Cdkn1a, Serpina3n, Cd68, Mmp12, Cd14, Comp, Col1a1, Lgals3*, and *Apoe*. The majority of these genes were linked to the functional pathways of neutrophils and monocyte/macrophage populations. Except for *Col1a1*, which was downregulated, all other genes demonstrated a significant increased expression in the CKD group (**Figures 1D–F**), suggesting their potential mechanistic involvement in vascular calcification during CKD progression.

### 3.2 Exploration of NETs-related mechanisms in aortic calcification in CKD

**Figure 2:**
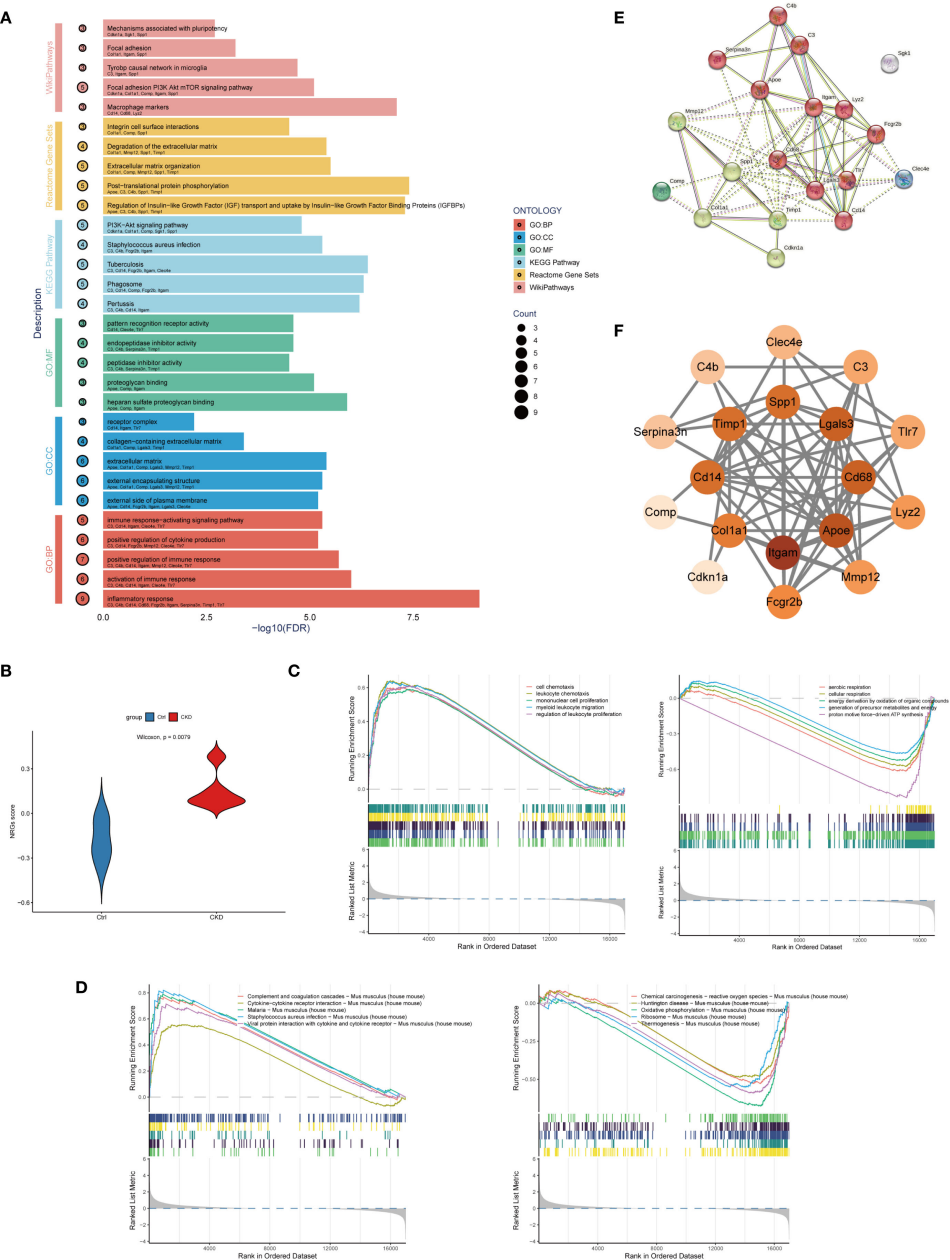
Functional analysis of NET-related candidate genes in CKD-associated vascular calcification. **(A)** Enrichment analysis of Gene Ontology (GO), KEGG, Reactome, and WikiPathways. The y-axis shows pathways, the x-axis shows statistical significance (–log10 FDR), and bubble size indicates gene count. **(B)** Violin plot comparing NET-related gene set variation analysis (GSVA) scores between CKD and control groups in the GSE146638 dataset (p < 0.01, Wilcoxon test). **(C)** Gene Set Enrichment Analysis (GSEA) based on GO: Biological Process (BP) terms in CKD and control samples. Top 5 enriched terms are shown. **(D)** GSEA based on KEGG pathways in CKD and control samples. Top 5 enriched terms are shown. **(E)** Protein–protein interaction (PPI) network constructed using the STRING database with a minimum interaction score of 0.4. The network includes 19 nodes and highlights extensive connectivity among NET-related genes. **(F)** Hub gene subnetwork visualized in Cytoscape using the cytoHubba plugin. Top-ranked genes based on degree centrality include Itgam, Apoe, Lgals3, Cd68, and Timp1.

To further explore the potential mechanisms linking NETs to aortic calcification in CKD, we performed functional enrichment analyses on the 19 previously identified NETs-related candidate genes. Pathway analyses were conducted using KEGG, Gene Ontology (GO), Reactome, and WikiPathways databases. Enrichment analysis indicated that these genes are involved in immune activation, inflammation, ECM reorganization, and signaling pathways that promote calcification, notably the PI3K-Akt-mTOR and IGF–IGFBP regulatory axes. These enriched pathways are biologically relevant to the pathogenesis of vascular calcification (**Figure 2A**).

To further assess the involvement of NETs-related genes in CKD-associated vascular pathology, we used Gene Set Variation Analysis (GSVA) to characterize pathway activity shifts. to the training dataset GSE146638. A NETs-related gene score (NRGs-score) was calculated for each sample, and differences between CKD and control groups were assessed using the Wilcoxon test. Analysis revealed a markedly higher NETs-related gene signature in CKD samples relative to the control group, indicating that NETs-associated gene expression is markedly enriched in CKD aortic tissue (**Figure 2B**).

Building upon the differential expression findings, We employed GSEA to analyze pathway enrichment using both GO: GO: BP and KEGG pathway databases to explore functional differences between CKD and control groups. GSEA revealed that CKD samples were significantly enriched in 505 GO: BP terms, whereas control samples were enriched in 85 GO: BP terms (**Figure 2C**). Furthermore, KEGG pathway analysis identified 25 significantly enriched pathways in the CKD group and 20 in the control group (**Figure 2D**), highlighting profound alterations in biological processes and signaling networks associated with CKD. Enrichment of GO and KEGG pathways in the CKD group revealed a pronounced activation of immune and inflammatory responses, particularly pathways involved in leukocyte chemotaxis, proliferation, and extracellular trap formation.

Based on the candidate genes identified through RobustRankAggreg (RRA) analysis, we constructed a protein–protein interaction (PPI) network using the STRING database, with the minimum required interaction score set to 0.4. The resulting interaction data were imported into Cytoscape (version 3.9.1) for visualization and topological analysis. The constructed network comprised 10 nodes and 69 edges, with a highly significant PPI enrichment p-value (< 1.0e−16), indicating non-random, biologically meaningful interactions among the selected genes. The top five hub genes ranked by network centrality were *Itgam*, *Apoe*, *Lgals3*, *Cd68*, and *Timp1*, suggesting their potential central roles in the NETs-associated signaling networks underlying CKD-related aortic calcification (**Figures 2E, F**).

### 3.3 Machine learning model to screen NETs-related key genes

**Figure 3:**
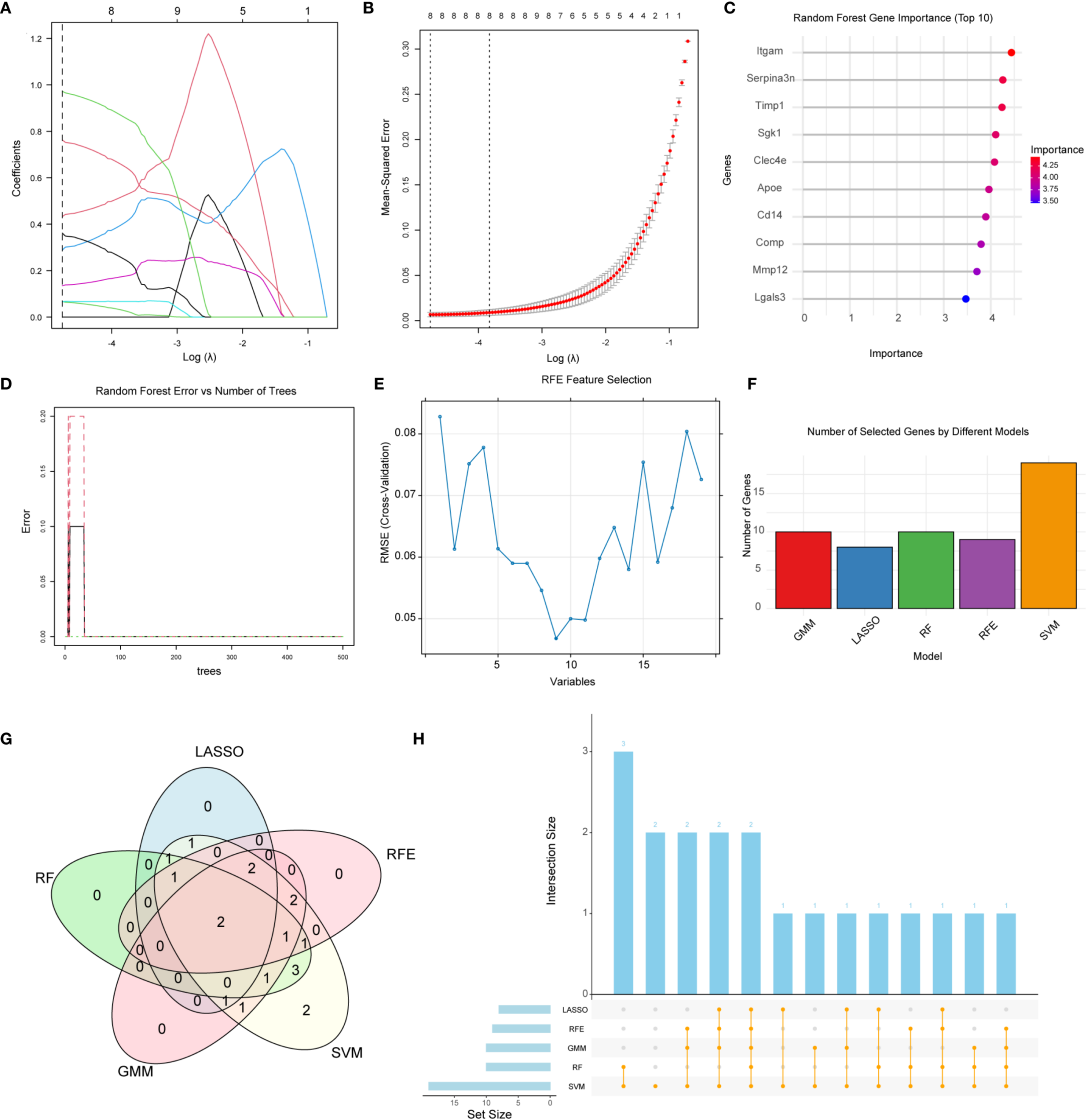
Identification of key NET-related genes by machine learning. **(A, B)** LASSO regression: **(A)** coefficient trajectories over log(λ); **(B)** 10-fold cross-validation for optimal λ. **(C)** Random Forest variable-importance scores; top genes exhibit strongest discrimination. **(D)** RF error-rate curve showing convergence with increasing trees. **(E)** SVM-based recursive feature elimination (RFE): RMSE versus retained variables. **(F)** Bar chart summarizing gene counts obtained by five algorithms (LASSO, RF, RFE, GMM, SVM). **(G)** Venn diagram displaying overlapping genes; Mmp12 and Comp were selected by all methods. **(H)** Upset plot illustrating intersections among gene sets across algorithms.

To identify robust candidate biomarkers associated with NETs-related mechanisms in CKD-associated aortic calcification, we applied five machine learning algorithms—Least Absolute Shrinkage and Selection Operator (LASSO), Random Forest (RF), Recursive Feature Elimination (32), Support Vector Machine (SVM), and Gaussian Mixture Modeling (GMM)—for feature selection. LASSO regression identified optimal lambda values using 10-fold cross-validation (**Figures 3A, B**), and selected a subset of genes with non-zero coefficients. RF analysis revealed stable model performance at approximately 100 trees (**Figure 3D**), and variable importance ranking highlighted several top candidates (**Figures 3C, D**). RFE evaluation further identified the optimal number of predictors based on cross-validation error (**Figure 3E**). Feature importance was also evaluated using a bar plot (**Figure 3F**), emphasizing consistently ranked top genes.

To integrate and compare the results across algorithms, a Venn diagram (**Figure 3G**), and UpSet plot (**Figure 3H**) were generated. Cross-method comparison revealed consistent gene overlaps among the different feature selection strategies, with two genes Mmp12 and Comp, consistently identified by all five algorithms.

### 3.4 Identification of core genes and exploration of biological functions

**Figure 4:**
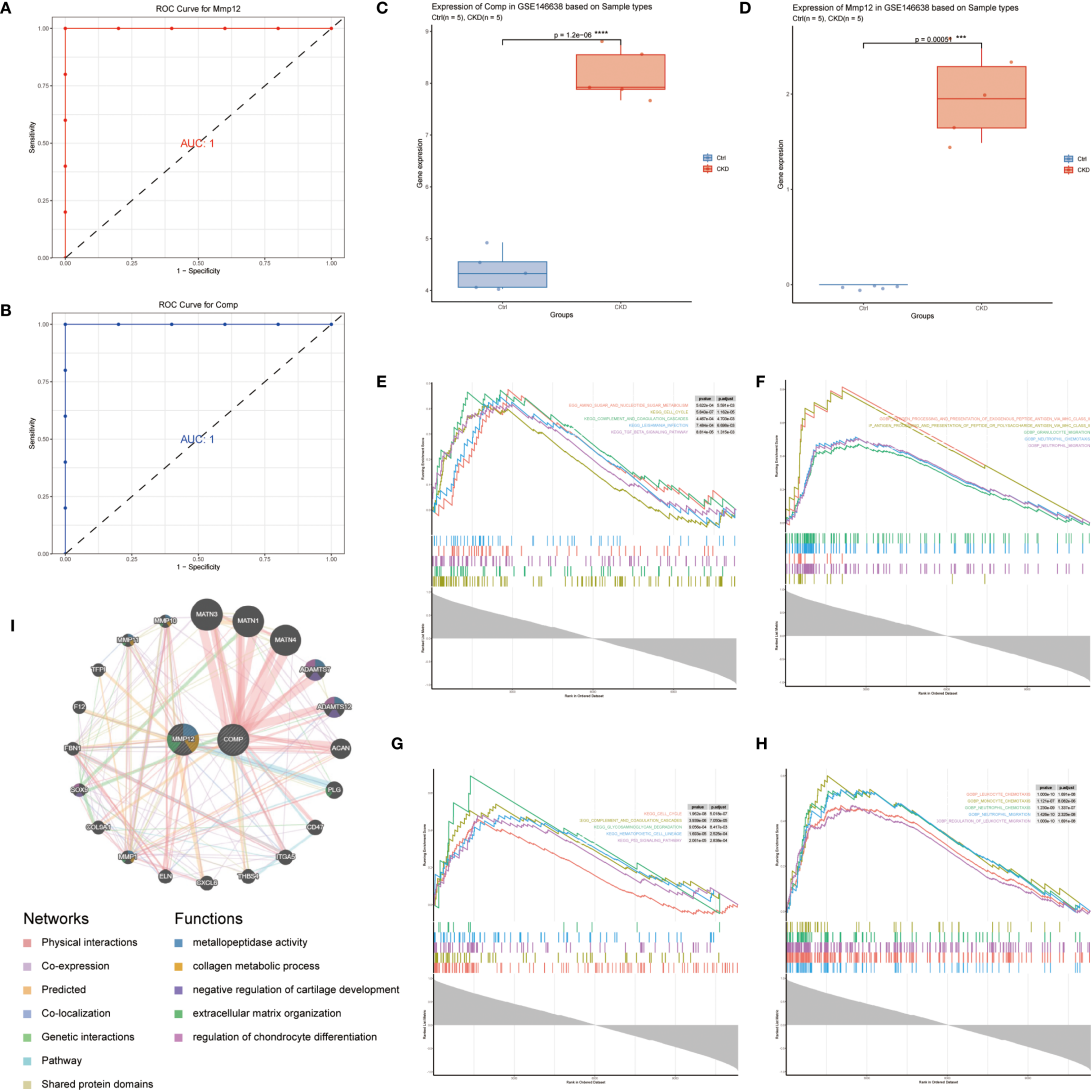
Diagnostic performance and functional characterization of key NET-related genes. **(A, B)** ROC curves for Mmp12 **(A)** and Comp **(B)** in the GSE146638 dataset; both yield AUC = 1.0. **(C, D)** Box plots showing significantly elevated Comp **(C)** and Mmp12 **(D)** expression in CKD samples versus controls (p < 0.001, Wilcoxon test). **(E–H)** GSEA results for top-ranked genes correlated with Comp **(E, F)** and Mmp12 **(G, H)**; KEGG pathways are on the left, GO: BP terms on the right. **(I)** GeneMANIA co-expression network illustrating functional interactions of Mmp12 and Comp with related genes (co-expression, shared pathways, and physical binding).

Using the GSE146638 dataset, we assessed the diagnostic value of the identified hub genes by constructing individual ROC curves for Mmp12 (**Figure 4A**) and Comp (**Figure 4B**). Both *Mmp12* and *Comp* showed strong ability to distinguish CKD samples from controls, showing area under the curve (AUC) scores approaching 1.0, indicating strong classification capability. Furthermore, expression levels of Mmp12 (**Figure 4D**) and Comp (**Figure 4C**) were significantly overexpressed in the CKD group when compared to the control group.

To explore the downstream functional implications of these genes, GSEA was performed based on high- versus low-expression stratification of *Mmp12* and *Comp*. KEGG and Hallmark gene sets revealed that both genes were associated with the activation of neutrophil chemotaxis and neutrophil migration pathways, suggesting their involvement in the inflammatory microenvironment of CKD-associated aortic calcification (**Figures 4E–H**).

Additionally, co-expression analysis using the GeneMANIA database identified genes with similar biological functions and expression profiles. Functional inference suggested that the gene network is heavily involved in ECM structural processes, further supporting the role of *Mmp12* and *Comp* in matrix remodeling and disease progression (**Figure 4I**).

### 3.5 Immunoregulatory landscape in aortic calcification in CKD

**Figure 5:**
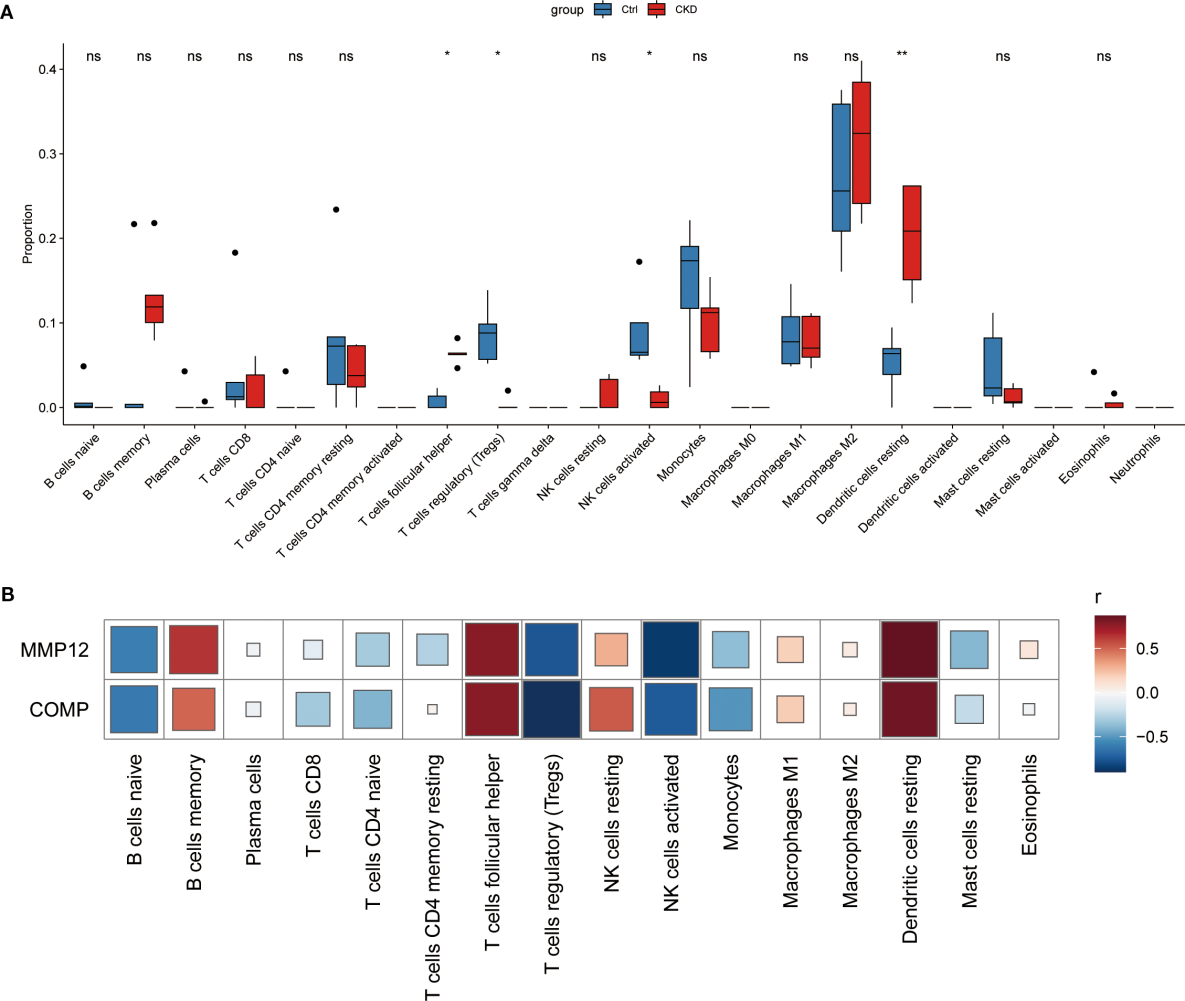
Immune-cell infiltration landscape of key NET-related genes in CKD-associated vascular calcification. **(A)** Relative abundance of 22 immune-cell types in CKD versus control samples, determined by CIBERSORT analysis of the GSE146638 dataset. **(B)** Spearman correlation between Mmp12/Comp expression and the abundance of infiltrating immune-cell subsets. * represents P<0.05, ** represents P<0.01, ns represents. P>0.05.

To characterize the immune microenvironment associated with CKD-related aortic calcification, we applied the CIBERSORT algorithm to the GSE146638 dataset to estimate the relative proportions of 22 immune cell types. Comparative analysis between CKD and normal samples revealed significant differences in the immune cell proportion, with dendritic cells (DCs) showing markedly elevated infiltration in the CKD group (**Figure 5A**). In addition, correlation analysis between the expression levels of the identified hub genes and immune cell scores demonstrated that cartilage oligomeric matrix protein (COMP) and matrix metalloproteinase 12(MMP-12) were significantly associated with multiple immune cell subsets, including memory B cells, regulatory T cells (Tregs), and dendritic cells (**Figure 5B**).

### 3.6 Molecular regulatory network and clinical correlation of core genes

**Figure 6:**
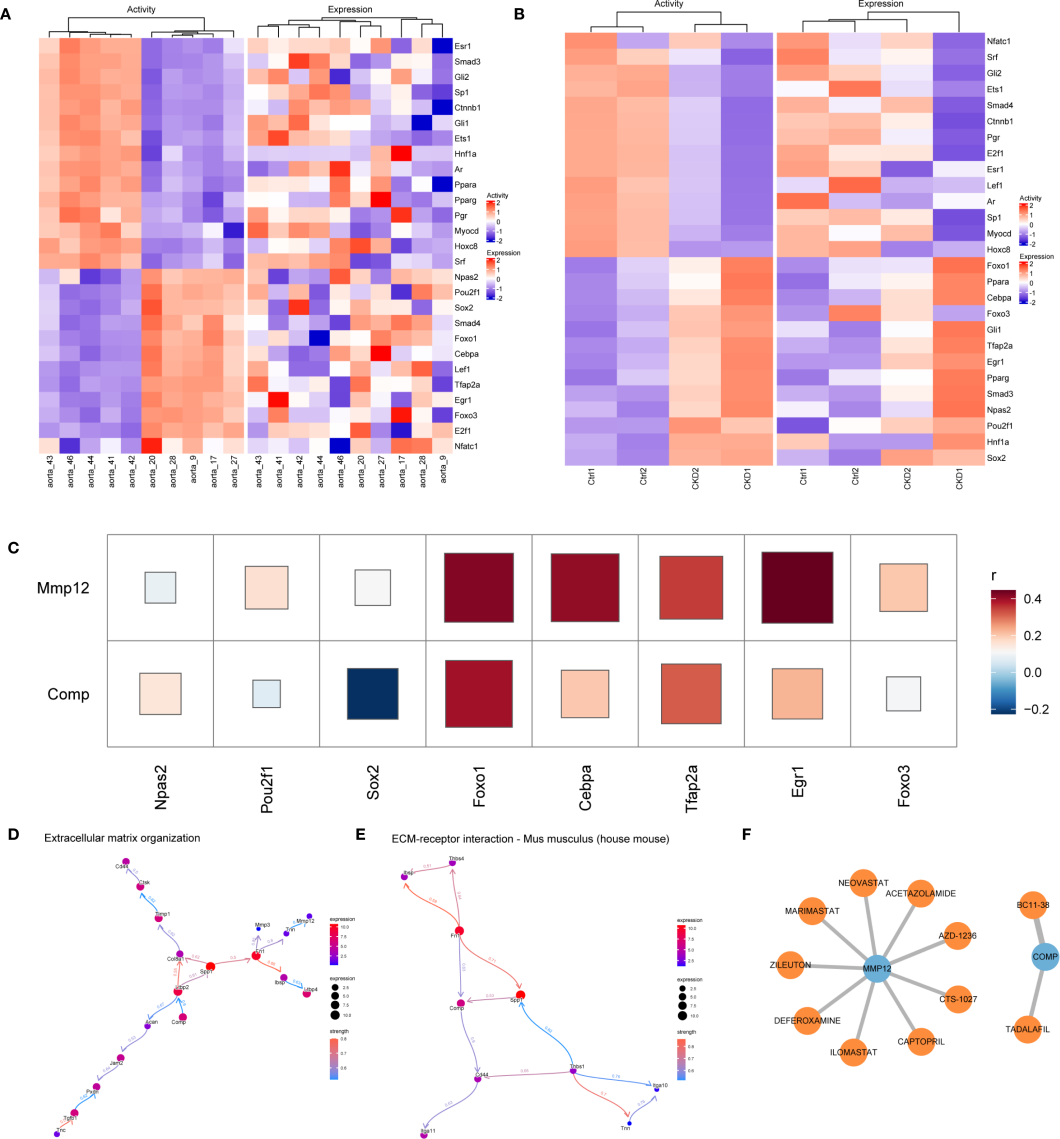
Regulatory networks and clinical relevance in CKD-related vascular calcification. **(A, B)** NETact-predicted transcription-factor (TF) activity and expression heatmaps in GSE146638 **(A)** and GSE159832 **(B)**. **(C)** Correlation heatmap showing positive associations of Foxo1 and Tfap2a with Mmp12 and Comp. **(D, E)** Bayesian networks (CBNplot) for genes co-expressed with Mmp12 **(D)** and Comp **(E)**. **(F)** Drug–gene interaction network (DGIdb, Cytoscape) highlighting Mmp12-targeting MMP inhibitors.

The upstream regulatory mechanisms of the hub genes were explored using the NETact algorithm to predict TF activity. A set of TFs—including *Npas2, Pou2f1, Sox2, Foxo1, Cebpa, Tfap2a, Egr1*, and *Foxo3* showed significant activation in CKD samples compared to controls (**Figures 6A, B**). Correlation analysis further revealed that *Foxo1* and *Tfap2a* were strongly associated with both *Mmp12* and *Comp* (**Figure 6C**), suggesting that they may serve as common upstream regulators.

Functional enrichment analysis was conducted based on genes exhibiting high correlation (r > 0.9) with *Comp* and *Mmp12*. GO and KEGG analyses highlighted their significant roles in matrix remodeling and receptor-mediated interactions. Additionally, GSEA based on expression ranking of *Comp* and *Mmp12* revealed enrichment in pathways related to neutrophil migration and immune signaling, highlighting their roles in inflammatory microenvironments. To further investigate causal regulatory relationships, a Bayesian network was constructed using the CBNplot algorithm. The analysis suggested that *Comp* may regulate *Ltbp2* within ECM-related pathways, while *Tnn* may act upstream of *Mmp12*. In the ECM–receptor interaction network, *Spp1* was predicted to modulate *Comp*, supporting its role in matrix remodeling during vascular calcification (**Figures 6D, E**).

Candidate therapeutic compounds were identified by integrating gene–drug interaction data from DGIdb and visualizing them in Cytoscape. Several inhibitors targeting MMP12, including Marimastat, Ilomastat, and AZD-1236 exhibited known anti-fibrotic or anti-inflammatory properties. Zileuton and Captopril also emerged as potential modulators, with established use in chronic inflammatory and cardiovascular conditions. Importantly, Tadalafil, a PDE5 inhibitor with clinical approval, was identified as a predicted interactor of COMP (**Figure 6F**).

### 3.7 IHC staining

**Figure 7:**
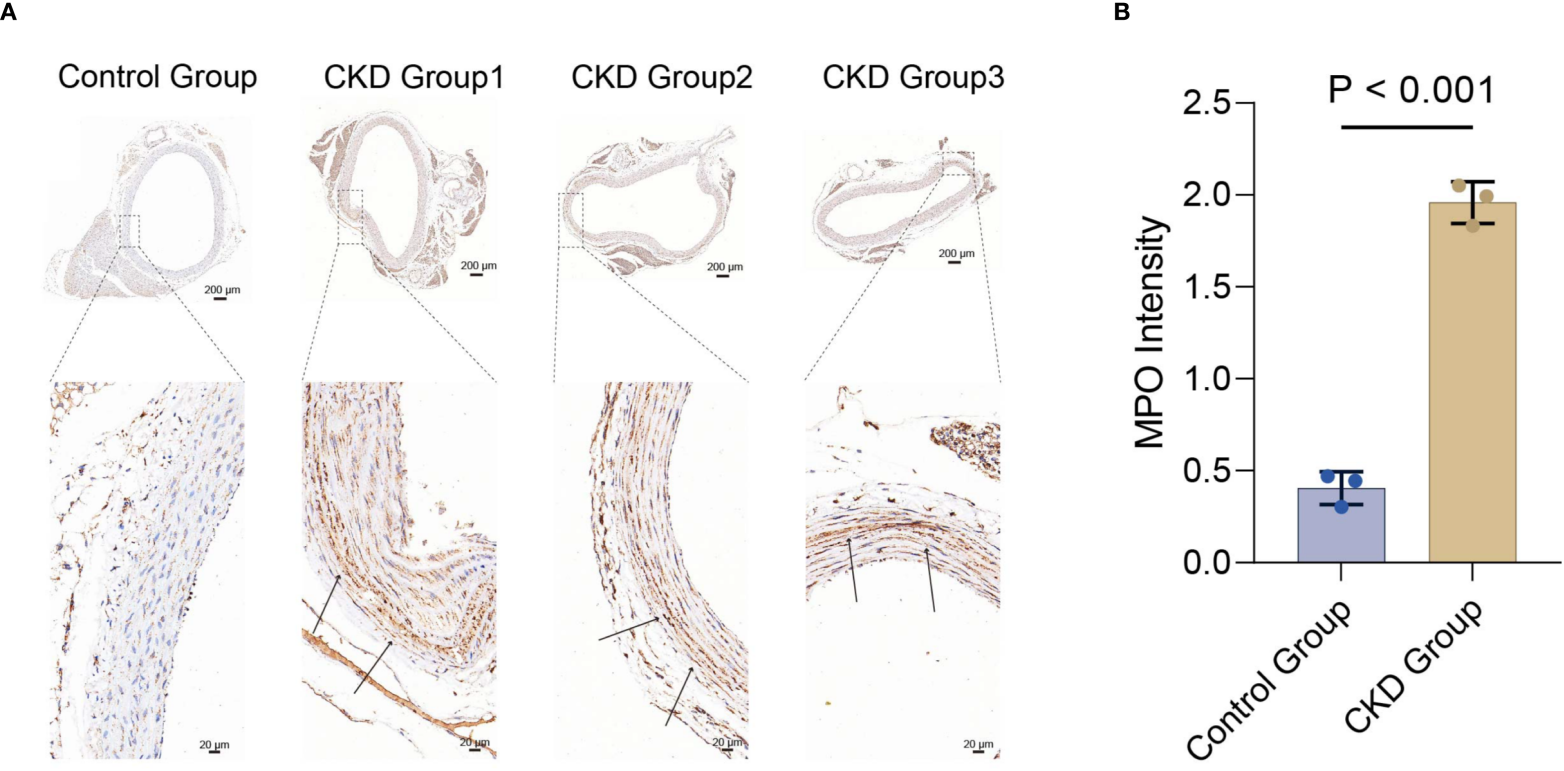
Immunohistochemical detection of MPO in vascular tissue from rat models. **(A)** Representative whole-section images (scale bar, 200 μm) and magnified views (scale bar, 20 μm) from control and CKD groups. **(B)** Quantitative analysis of MPO immunostaining intensity; data are presented as mean ± SD (*p* < 0.001 vs. control), n=3 animals. MPO, myeloperoxidase.

We attempted to perform IHC staining for MPO in the CKD model rats(n=3) and controls(n=3). IHC staining revealed that MPO levels in the rat thoracic aorta were significantly higher in the CKD group than in the control group(*p* < 0.001)(**Figures 7A, B**).

### 3.8 qRT-PCR and Western blot

qRT-PCR and Western blotting were performed to evaluate the expression of MMP-12 and COMP in the model rats and controls.

**Figure 8:**
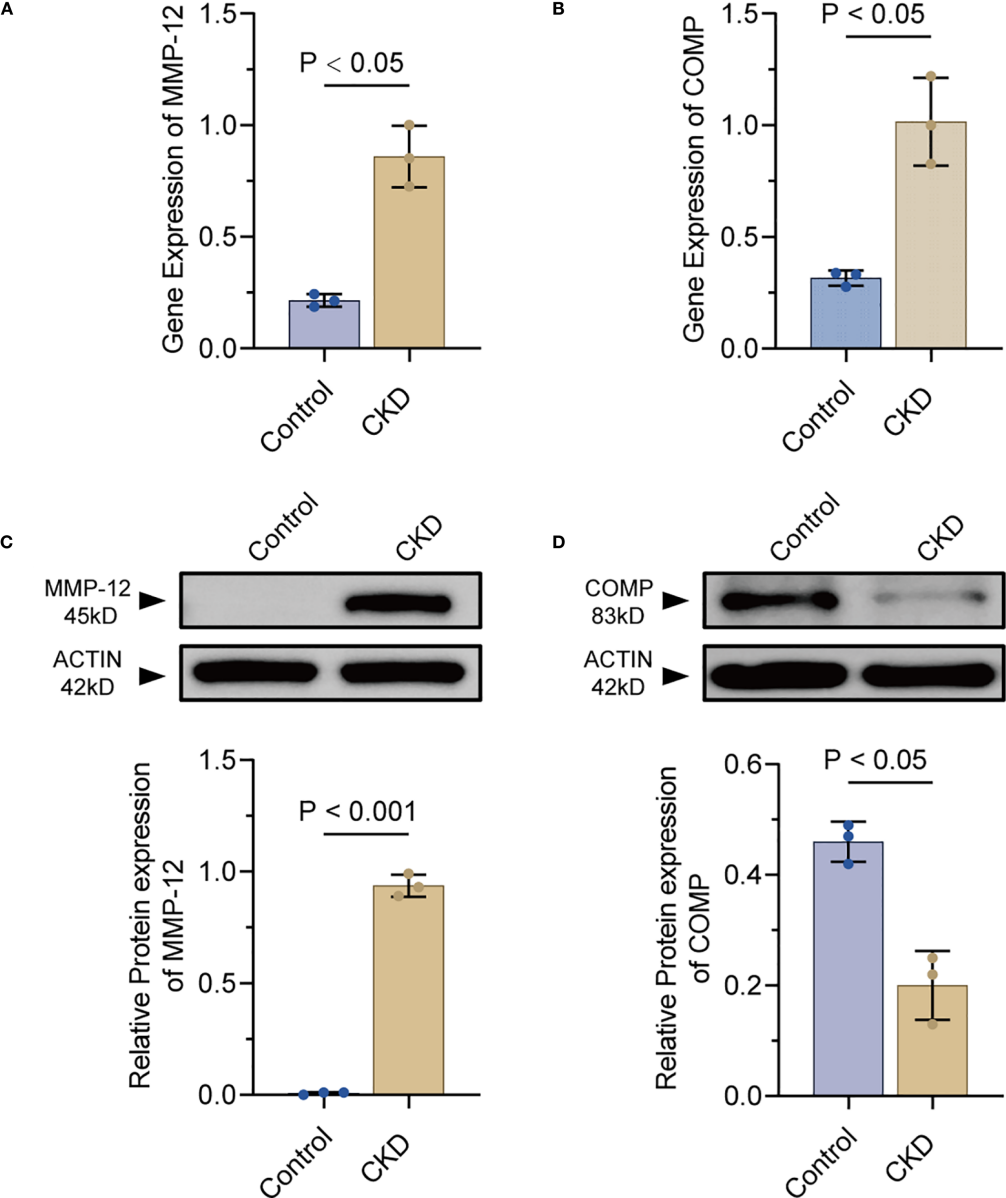
Experimental validation of MMP-12 and COMP expression in rat aortae(n=3). **(A, B)** qPCR quantification of MMP-12 and COMP mRNA in CKD versus control rat aortae. **(C, D)** Representative Western blot bands and densitometry for MMP-12 and COMP proteins in CKD and control groups. MMP-12, matrix metalloproteinase-12; COMP, cartilage oligomeric matrix protein.

qRT-PCR confirmed statistically distinct expression patterns of *Mmp-12* and *Comp* between the two groups between the CKD group and the control group (*p* < 0.05)*(* **Figures 8A, B**).

Western blotting analysis revealed the expression of MMP-12 was notably upregulated in the CKD group compared to the control group (*p* < 0.05, **Figure 8C**). In contrast, the levels of COMP were significantly decreased in the CKD group relative to the control group *(p< 0.05*, **Figure 8D**). These findings not only validated previous findings, but also revealed the important roles of MMP-12 and COMP in the pathological processes underlying vascular calcification in rodent models.

## 4 Discussion

In individuals with CKD, vascular calcification (VC) is a common complication that markedly increases cardiovascular risk. Accumulating evidence has linked VC progression to persistent inflammation, largely driven by cytokines including TNF-α, IL-1β, and IL-6 (33). NET formation is an inflammatory cell death mechanism in neutrophils, characterized by the release of chromatin and other nonspecific components (34). Hyperactivation of NETs may provoke dysregulated inflammation and tissue injury, primarily due to the accumulation of extracellular chromatin and pro-inflammatory mediators like TNF-α and IL-1β (35, 36). Studies have demonstrated the presence of NETs in both human and experimental murine abdominal aortic aneurysms (AAAs) (37). Specifically, neutrophils undergo NETosis during the early phase of AAA growth, and this process requires IL-1β, MMP-2, and MMP-9 within neutrophils (38). Importantly, these members of the NETs-related family also play an important role in the development of another cardiovascular disease—VC (33). Our validation experiments further demonstrated that MPO expression was markedly elevated in the thoracic aorta of rats with CKD. The precise molecular pathways through which NETs influence the development and progression of VC in CKD remain to be fully elucidated. The expanding use of high-throughput microarrays and computational analyses has enhanced the ability to identify genes implicated in VC.

To dissect the involvement of NETs-linked genes in CKD-related vascular calcification, we utilized a comprehensive multi-omics approach, integrating bioinformatics tools with machine- learning-based gene prioritization, immune cell profiling, transcriptional regulatory inference, and drug–gene interaction mapping. We performed differential gene expression analyses on two independent RNA-sequencing datasets derived from rodent models of CKD, leading to the identification of 19 robust NETs-related candidate genes. Enrichment analysis indicated that these genes were primarily associated with immune-inflammatory pathways and ECM remodeling, supporting the mechanistic involvement of NETs in CKD-related vascular calcification. Among the identified candidate genes, *Mmp2* and *Comp* emerged as the most consistently selected key biomarkers across all five machine learning algorithms. Both genes were significantly upregulated in CKD samples from rat and mouse, and exhibited excellent diagnostic performance, with area under the curve (AUC) values exceeding 0.95.

MMP-12, a macrophage-secreted elastase, mediates elastolytic degradation of ECM components and drives pathological tissue remodeling (39). Recent genomic analyses identified *Mmp12* as the most significantly upregulated gene in stenotic aortic valve cusps (40). It has been reported that immunohistochemical analysis localized MMP-12 protein to regions of advanced calcification. Mechanistically, MMP-12 promotes RUNX-2 and BMP-2 expression, enhances alkaline phosphatase (ALP) activity, and accelerates calcium deposition via activation of the p38 MAPK-LRP-6/β-catenin signaling axis (41). These findings align with growing evidence that MMP-12 critically regulates vascular calcification through ECM destabilization. Consistent with prior observations, our experiments confirmed elevated MMP-12 expression in thoracic aorta of CKD rats. We propose a pathogenic cascade wherein macrophage infiltration in chronic inflammatory microenvironments induce *Mmp12* overexpression, thereby initiating a feed-forward loop of ECM degradation and ectopic calcification.

As a vascular extracellular matrix glycoprotein, COMP acts as a natural inhibitor of vascular calcification. Experimental studies demonstrate that COMP expression is significantly downregulated in mineralized VSMCs and arterial tissues. Mechanistically, Via its C-terminal domain, COMP binds directly to bone morphogenetic protein-2 (BMP-2), thereby interfering with BMP-2 receptor engagement and inhibiting subsequent osteochondrogenic signaling (41). Notably, macrophages lacking *Comp* exhibit pro-atherogenic and osteogenic phenotypes via integrin β3-dependent signaling, exacerbating atherosclerotic calcification (42). These findings collectively establish COMP as an essential modulator of vascular calcification development. Interestingly, our validation studies confirmed reduced COMP protein levels in CKD rat models, consistent with prior clinical observations. However, integrated bioinformatics analyses and experimental validation revealed a paradoxical upregulation of *Comp* mRNA in CKD rats. We hypothesize that this transcriptional discordance may result from compensatory feedback mechanisms triggered by sustained COMP protein depletion.

Our functional enrichment analyses (GO, KEGG, Hallmark) further revealed that both *Mmp12* and *Comp* are enriched in pathways related to neutrophil chemotaxis, cell migration, and ECM organization, suggesting their participation in immune-mediated vascular calcification processes. In addition, TF activity analysis identified *Foxo1* and *Tfap2a* as potential shared upstream regulators of both genes. Notably, previous research has demonstrated that MMP-12 can promote neutrophil polarization through activation of the **F**OXO1 signaling axis, leading to a pro-apoptotic phenotype (43). Together, these findings provide a mechanistic framework to further explore NET-associated pathways within the framework of CKD-associated vascular calcification, and highlight a coordinated transcriptional regulatory network that may underlie CKD-related vascular pathology.

Importantly, our drug–gene interaction analysis identified several compounds with potential therapeutic relevance. Specifically, MMP-12 was associated with a group of known MMP inhibitors (Marimastat, Ilomastat, AZD-1236) as well as anti-inflammatory agents (Zileuton, Captopril). Among them, Marimastat is a broad-spectrum matrix metalloproteinase (MMP) inhibitor capable of suppressing multiple MMPs, including MMP-1, MMP-2, MMP-7, MMP-9, and MMP-12. Notably, in mouse models, Marimastat has demonstrated the ability to inhibit MMP-12–induced inflammatory responses, reduce neutrophil and macrophage recruitment, and lower levels of pro-inflammatory cytokines (19, 44). Meanwhile, Comp was found to be associated with the clinically approved PDE5 inhibitor Tadalafil. In a case report, Tadalafil was administered to treat penile calcification lesions in a patient with end-stage renal disease, which resulted in a favorable clinical response. This finding suggests a novel potential role for Tadalafil in ameliorating vascular calcification in CKD patients (45). Taken together, these findings highlight a plausible pharmacological strategy to modulate NETs-related gene activity and mitigate aortic calcification in CKD. Furthermore, these data indicate that modulation of NET-related gene activity through repurposed, clinically available agents may attenuate aortic calcification in CKD. The established safety profiles of several of these compounds further support the feasibility of rapid translational evaluation.

However, there are also several limitations in our study. First, the analyses were based on publicly available transcriptomic data from rodent models, which may not fully recapitulate the complexity of human CKD pathology. Second, while our integrative approach prioritized candidate regulators and therapeutic agents, functional confirmation using *in vivo* and *in vitro* experimental models is warranted to confirm their mechanistic roles and therapeutic efficacy. Lastly, the small sample size in one of the datasets (GSE159833) may limit statistical power, and future validation in larger, independent cohorts is important.

## 5 Conclusion

In summary, this study maps the molecular contribution of NET-related genes to CKD-driven aortic calcification in rodent models. Through multi-omics data and machine learning algorithms, we pinpoint *Mmp12* and *Comp* as key regulators of immune-mediated ECM remodeling and vascular pathology. Both genes demonstrated robust diagnostic potential and were found to be transcriptionally regulated by *Foxo1* and *Tfap2a*. Importantly, our network-based drug screen nominates Marimastat and tadalafil as readily available agents capable of attenuating NET-driven calcification. These findings offer both mechanistic insight and a practical starting point for future biomarker studies and NET-targeted treatments in CKD-related vascular disease.

## Data Availability

The original contributions presented in the study are included in the article/supplementary material. Further inquiries can be directed to the corresponding author.
